# Update on the management of older patients with pancreatic adenocarcinoma: a perspective from medical oncology

**DOI:** 10.1007/s12094-024-03386-8

**Published:** 2024-02-08

**Authors:** Mónica Guillot Morales, Laura Visa, Elena Brozos Vázquez, Jaime Feliu Batlle, Parham Khosravi Shahi, Berta Laquente Sáez, Borja López de San Vicente Hernández, Teresa Macarulla, Regina Gironés Sarrió

**Affiliations:** 1 Spanish Society of Medical Oncology (SEOM) Oncogeriatrics Section, Department of Medical Oncology, Son Espases University Hospital, Carretera de Valldemossa, 79, Islas Baleares, 07120 Palma de Mallorca, Spain; 2 Spanish Society of Medical Oncology (SEOM) Oncogeriatrics Section, Mar-Parc de Salut Mar Hospital, Barcelona, Spain; 3 Spanish Society of Medical Oncology (SEOM) Oncogeriatrics Section, A Coruña University Clinical Hospital, A Coruña, Spain; 4 Multidisciplinary Spanish Group of Digestive Cancer (GEMCAD), La Paz University Hospital, IDIPAZ, CIBERONC, Cathedra UAM-AMGEN, Madrid, Spain; 5 Spanish Society of Medical Oncology (SEOM) Oncogeriatrics Section, Gregorio Marañón University Hospital, Madrid, Spain; 6 Spanish Society of Medical Oncology (SEOM) Oncogeriatrics Section, ICO L´Hospitalet-IDIBELL, Barcelona, Spain; 7 Spanish Society of Medical Oncology (SEOM) Oncogeriatrics Section, Basurto University Hospital, Bilbao, Spain; 8 Spanish Cooperative Group for the Treatment of Digestive Tumours (TTD), Hebron University Hospital, Vall d, Barcelona, Spain; 9 Spanish Society of Medical Oncology (SEOM), Polytechnic la Fe University Hospital, Valencia, Spain

**Keywords:** Pancreatic cancer, Older patient, Chemotherapy, Geriatric assessment

## Abstract

In the context of pancreatic cancer, surgical intervention is typically recommended for localized tumours, whereas chemotherapy is the preferred approach in the advanced and/or metastatic setting. However, pancreatic cancer is closely linked to ageing, with an average diagnosis at 72 years. Paradoxically, despite its increased occurrence among older individuals, this population is often underrepresented in clinical studies, complicating the decision-making process. Age alone should not determine the therapeutic strategy but, given the high comorbidity and mortality of this disease, a comprehensive geriatric assessment (CGA) is necessary to define the best treatment, prevent toxicity, and optimize older patient care. In this review, a group of experts from the Oncogeriatrics Section of the Spanish Society of Medical Oncology (*Sociedad Española de Oncología Médica*, SEOM), the Spanish Cooperative Group for the Treatment of Digestive Tumours (*Grupo Español de Tratamiento de los Tumores Digestivos*, TTD), and the Multidisciplinary Spanish Group of Digestive Cancer (*Grupo Español Multidisciplinar en Cáncer Digestivo*, GEMCAD) have assessed the available scientific evidence and propose a series of recommendations on the management and treatment of the older population with pancreatic cancer.

## Introduction 

In Spain, pancreatic cancer is estimated to be the ninth to tenth most common cancer in both sexes [1], with an estimate of 4770 new incident cases in 2023. Its prevalence is low, but it is one of the main causes of death from cancer, with an estimated survival of 6–7 months [1]. Biliopancreatic malignancies are correlated with age [2]. The incidence of pancreatic cancer increases with age, being 72 years the average age at diagnosis. Twenty-five percent of diagnoses are made at 65–74 years, 29% at 75–84 years, and 13% at > 85 years [3, 4]. Ageing is a heterogeneous process, so the management of the older population with pancreatic cancer requires a multidimensional approach, making it essential to incorporate geriatric tools in decision-making for this population [5].

To better manage the older population with pancreatic cancer, a group of medical oncologists from the Oncogeriatrics Section of the Spanish Society of Medical Oncology (*Sociedad Española de Oncología Médica*, SEOM), the Spanish Cooperative Group for the Treatment of Digestive Tumours (*Grupo Español de Tratamiento de los Tumores Digestivos*, TTD), and the Multidisciplinary Spanish Group of Digestive Cancer (*Grupo Español Multidisciplinar en Cáncer Digestivo,* GEMCAD) have carried out an exhaustive review of the available scientific evidence and have proposed a series of recommendations on the management and treatment of the older population with pancreatic cancer. Information was extracted from clinical studies including older patients with PDAC from PubMed between the period of 2007 to 2023. In accordance with the literature review performed, the age of 65 or 70 was considered as a threshold to categorize older patients.

## The role of comprehensive geriatric assessment in patient selection 

Older patients with pancreatic ductal adenocarcinoma (PDAC) are underrepresented in clinical trials that support clinical guidelines and establish the most beneficial treatments. External validation of clinical guidelines that recommend surgery in localized tumours and chemotherapy in advanced and metastatic tumours is limited in the older population, especially in treatment with curative intent [6].

To date, SEOM recommends including the geriatric assessment (GA) in the therapeutic decision-making process [5]. This approach has demonstrated that almost 90% of selected patients over 70 years of age with PDAC may benefit from the same standard treatment as younger patients [7].

The comprehensive geriatric assessment (CGA) is a multidomain assessment that collects major functional variables, such as autonomy in the performance of basic and instrumental activities of daily life, mental health, physical state, nutritional state, comorbidity, the presence of geriatric syndromes (e.g., incontinence, hearing loss, falls in the last 6 months), social support, and polypharmacy. It allows a complete initial evaluation and the establishment of a care plan to optimize the interventions in patients > 70 years of age. In the patient with PDAC, a correct nutritional evaluation and a support plan in this regard becomes more important, both for curative and palliative approach.

Up to 25% of patients with PDAC present frailty, according to the Fried criteria, which is a factor of poor prognosis (Table 1) [8]. In frail patients, oncogeriatric interventions have been shown to reduce surgical complications and improve compliance with chemotherapy, with fewer modifications to the assigned regimen and a lower incidence of toxicity [9].

**Table 1:** Fried Frailty Index [86]

Fried Frailty Index	Positive
Weight loss	> 5 kg unintentional lost in the previous year or 5% of the weight of the patient under follow-up
Weakness, fatigue	In the last week how often do you feel that any activity is an effort or that you cannot continue? 3–4 days or almost all the time
Physical activity	Reduction of weekly physical activity Men: < 383 kcal/week Women: < 270 kcal/week
Walking speed in 4.5 m	Men ≤ 173 cm: ≥ 7 s > 173 cm: ≥ 6 s
	Women ≤ 159 cm: ≥ 7 s > 159 cm: ≥ 6 s
Grip strength	Men BMI ≤ 24: ≤ 29 BMI 24.1–26: ≤ 30 BMI 26.1–28: ≤ 31 BMI > 28: ≤ 32
	Women BMI ≤ 23: ≤ 17 BMI 23.1–26: ≤ 17.3 BMI 26.1–29: ≤ 18
Frailty ≥ 3 criteria Intermediate frailty: 1–2 criteria Non-frailty older patient: 0 criteria	

*BMI* body mass index

A complete CGA should be performed in coordination with specialist geriatricians, but not all hospitals have such staff. In most centres, faster screening is performed with the G8 scale or the Vulnerable Elders Survey (VES-13), which allows the selection of patients who would most benefit from an extended CGA. However, these scales in isolation are not powerful enough to predict undesirable serious adverse events [10].

**Fig. 1:**
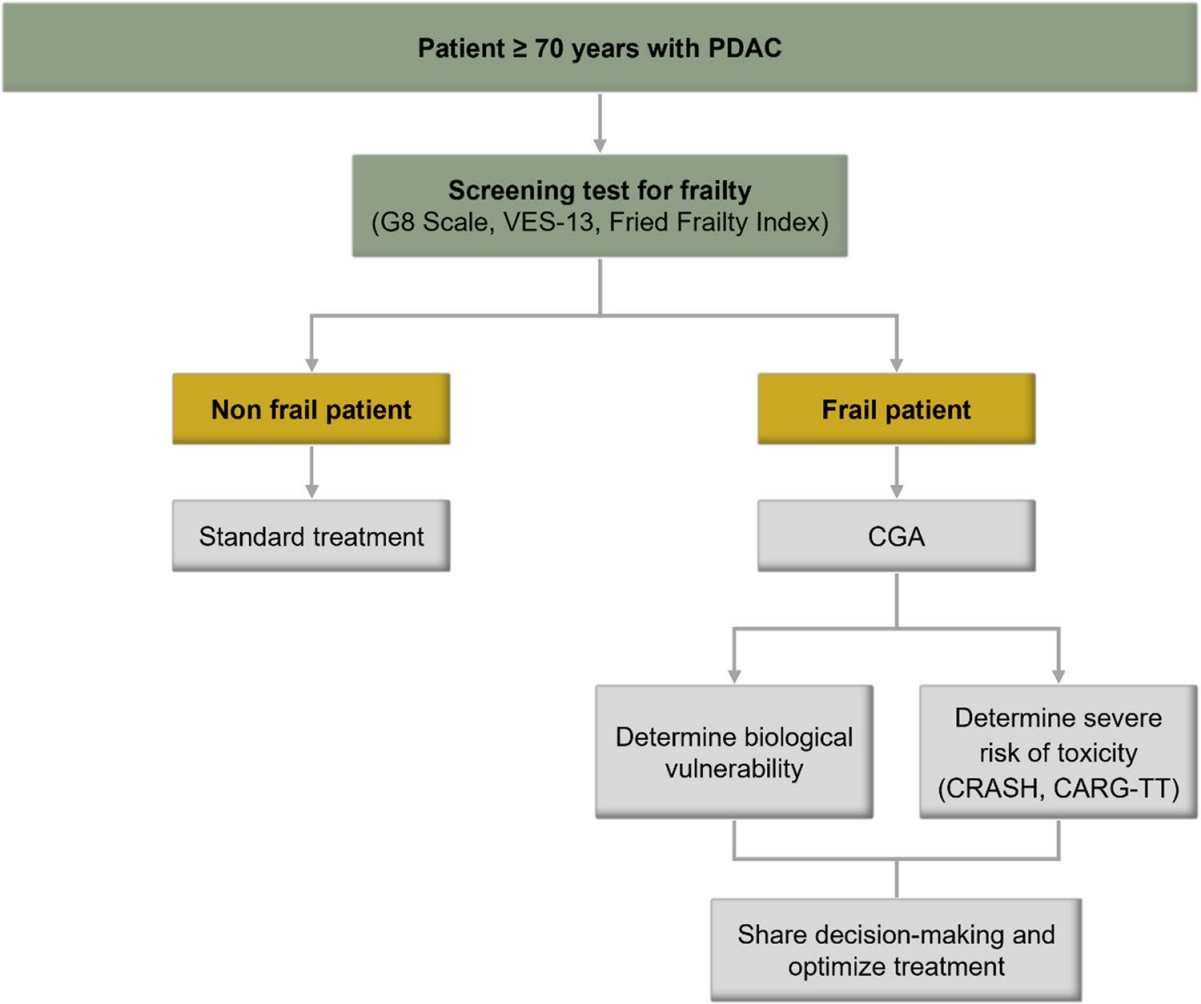
Tools for the evaluation of older patients with pancreatic cancer. *G8* Geriatric-8, *VES-13* vulnerable elders survey-13, *CRASH* chemotherapy risk age scale for high-age patients, *CARG-TT* Cancer and Aging Research Group chemotherapy toxicity tool

Other easily accessible and complete tools can improve the evaluation of older patients with PDAC, such as the Cancer and Ageing Research Group Toxicity Tool (CARG-TT) and the Chemotherapy Risk Assessment Scale for High-Age Patients (CRASH), by taking into account the analytical results, the selected treatment, and the extent of the disease, to predict the incidence of toxicity [11, 12] (Fig. 1). Other tools predict early mortality, unexpected hospitalization, or postsurgical complications [13–15].

## Resectable and borderline-resectable pancreatic adenocarcinoma

### Surgical treatment

Only 15–20% of patients have resectable PDAC at diagnosis. In this scenario, surgery is the standard treatment. Its objective is to achieve complete resection (R0) with disease-free resection margins.

PDAC surgery will be a cephalic duodenopancreatectomy in tumours in the head of the pancreas or a distal or total pancreatectomy in tumours in the tail or body of the pancreas. These are highly complex surgeries that should preferably be performed in centres with a high volume of patients (> 20 pancreatic surgeries per year) [16]. Despite the improvements achieved in surgical techniques, intensive care, pre- and postsurgery prehabilitation programs, and patient selection, these surgeries carry a high mortality rate (approximately 2–5%) [17] and a high comorbidity rate (approximately 40–60%) [18]. The influence of age on long-term oncological outcomes following pancreatic cancer resection remains uncertain because of the variety of factors that can influence surgery. According to several recent retrospective studies, older patients do not develop higher rates of surgical complications or higher mortality at 90 days than younger patients, although they do have a longer mean hospital stay [19–21]. In a prospective Dutch trial that compares 198 patients ≥ 75 years with 638 patients < 75 years, the median overall survival (OS) was 15,0 months compared with 21,0 months (p < 0.001), respectively. However, only a third of patients ≥ 75 years received adjuvant chemotherapy, which might explain the shorter long-term survival [21]. In older patients, it is especially important to start a prehabilitation program before surgery.

### Adjuvant treatment

After optimal cancer surgery, 80% of patients relapse within 2 years. Adjuvant chemotherapy has been shown to provide a significant OS benefit in all PDAC patients treated with curative surgery who have an Eastern Cooperative Oncology Group (ECOG) score of 0–1 and good nutritional status. Adjuvant treatment should start within 12 weeks after surgery. Receiving full adjuvant treatment is an independent factor of OS [22]. These data are relevant since 50% of the operated patients do not receive or do not complete the planned adjuvant treatment, due to either postsurgical complications, tumour progression, or adverse events of the treatment, which percentage rises to 70% in patients ≥ 70 years old [21, 23, 24]. In randomized clinical trials that have shown a benefit in OS of adjuvant chemotherapy, patients ≥ 70 years old are underrepresented, and the few who are included do not represent the majority of cases seen in daily clinical practice, as they are patients who meet very restrictive inclusion criteria (Table 2).

**Table 2:** Percentage of older patients included in the most relevant PDAC adjuvant clinical trials

Study N	Age, median (range)	Elderly	Treatment	Completion full treatment	OS months (95% CI)	5 yr-OS
CONKO-001 [22, 25] Phase III, *N* = 354	62 (34–82)	62% (≥ 65 yr)	A: Observation B: Gemcitabine	A: NR B: 62%	A: 20.2 (17–23.4) B: 22.8 (18.4–25.8)	A: 10.4% B: 20.7%
ESPAC-3 [26] Phase III, *N* = 1088	63 (31–85)	NR	A: 5FU + Leucovorin B: Gemcitabine	A: 55% B: 60%	A: 23 (21.1–25) B: 23.6 (21.4–26.4)	A: 15.9% B: 17.5%
ESPAC-4 [27] Phase III, *N* = 730	65 (37–81)	30% (≥ 65 yr)	A: Gemcitabine B: Gemcitabine + capecitabine	A: 65% B: 54%	A: 25.5 (22.7–27.9) B: 28 (23.5–31.5)	A: 24.4% B: 28.8%
APACT [28] Phase III, *N* = 866	64 (34–86)	45% (≥ 65 yr)	A: Gemcitabine B: Gemcitabine + nab-paclitaxel	A: 71% B: 66%	A: 37.7 (NA) B: 41.8 (NA)	A: 31% B: 38%
PRODIGE 24 [29, 30] Phase III, *N* = 493	63 (30–79)	21% (≥ 70 yr)	A: Gemcitabine B: mFOLFIRINOX	A: 79% B: 66%	A: 35.5 (30.1–40.3) B: 53.5 (43.5–58.4)	A: 31.4% B: 43.2%

*5-FU* 5-Fluorouracil; mFOLFIRINOX: modified leucovorin calcium (folinic acid), fluorouracil, irinotecan hydrochloride, and oxaliplatin, *NA* not available, *NR* not reported, *OS* overall survival, *PDAC* pancreatic ductal adenocarcinoma, *yr* year

Of the first phase III studies, the CONKO-001 study stands out. In it, 368 patients (62% ≥ 65 years old) were randomized to receive adjuvant treatment with gemcitabine or observation after curative-intent surgery. Adjunctive gemcitabine significantly increased progression-free survival (PFS) versus observation (13.4 vs. 6.9 months; hazard ratio [HR] 0.55; *p* < 0.001), regardless of lymph node involvement and resection margins. No difference by age was observed in the multivariate analysis (*p* = 0.06). The gemcitabine arm slightly improved OS (22.8 vs. 20.2 months), reaching a 10-year survival rate of 12.2% compared to 7.7% in the control arm. Gemcitabine treatment was well tolerated. The most frequent toxicities were haematological (anaemia and leukopenia), digestive (nausea and vomiting), and hepatological (elevated liver enzymes). Grade 3–4 toxicity was rare [22, 25]. A later study evaluated whether adjuvant treatment with 5-fluorouracil (5-FU) was superior to gemcitabine after resection in patients with PDAC. No significant differences were detected in OS between the two arms (23.0% vs. 23.6%; *p* = 0.039). However, in the 5-FU arm, a higher incidence of grade 3–4 toxicity was observed than in the gemcitabine arm (14.0% vs. 7.5%) in the form of mucositis, diarrhoea, and hospitalizations. The percentage of included patients ≥ 65 years old was not specified, but as in the above study, age was not significantly associated with prognosis [26].

More recently, the results of the ESPAC-4 study have been published. It included 730 patients, of whom 30% were ≥ 65 years old. They were randomized to receive gemcitabine with capecitabine (GemCap) versus gemcitabine alone. A modest but significant benefit in OS was demonstrated for the arm treated with GemCap (28.8 vs. 25.5 months; HR 0.82; *p* = 0.032). The benefit of the combination was consistent in all subgroups of patients analysed, including patients ≥ 65. The maximum benefit of the combined treatment was observed in patients with tumours < 30 mm, without local invasion and with R0 surgery. No significant differences were established in the incidence of serious adverse events between both arms, despite higher incidences of diarrhoea, neutropenia, and hand–foot syndrome in the arm treated with the combination [27]. Based on these results, international clinical guidelines recommend the GemCap regimen for the adjuvant treatment of PDAC patients, which, like gemcitabine alone, is a good option for the adjuvant treatment of selected older patients. Similar results were obtained in the APACT phase III trial when nab-paclitaxel was added to gemcitabine [28].

Finally, the PRODIGE 24 study randomized 493 patients to receive leucovorin (folinic acid), 5-FU, irinotecan, and oxaliplatin (FOLFIRINOX) administered in 12 biweekly cycles of the modified no-bolus regimen of 5-FU or gemcitabine in monotherapy. Twenty percent of the patients included were ≥ 70 years old, with a maximum age of 79 years. The included patients were well selected, with good prognostic factors. The study demonstrated a significant increase in PFS in the FOLFIRINOX arm versus gemcitabine (21.4 vs. 12.8 months; HR 0.66; *p* < 0.001). OS was 53.5 months versus 35.5 months (HR 0.68; *p* = 0.001), with a 5-year survival rate of 43.2% versus 31.4%, these being the best survival data published thus far. In the multivariate analysis, age < 70 years was a favourable prognostic factor for survival. Only 66% of patients received the whole planned 12 cycles of FOLFIRINOX, compared to 80% of patients who received all of the gemcitabine, largely due to toxicity. The triplet arm presented significantly higher grade 3–4 toxicity than the gemcitabine arm (75.5% vs. 51.1%), neutropenia, diarrhoea, mucositis, neurotoxicity, and fatigue being the most frequent toxicities. Regarding age, no significant increase in adverse events was observed in older patients [29, 30]. Due to the small representation of older patients in this study and the wide spectrum and degree of adverse events, the FOLFIRINOX scheme is not routinely recommended in patients ≥ 70 years old. If this chemotherapy regimen is considered for any patient, it is recommended to perform a CGA first to measure the frailty of the patient and to consider the administration of primary prophylaxis with granulocyte colony-stimulating factor [31].

It can be summarized that although there are no conclusive data in the older population, age does not seem to be a determining factor in deciding whether to administer adjuvant chemotherapy. Older patients with good functional status after surgery and without comorbidities can be treated preferably with gemcitabine-based regimens. The administration of FOLFIRINOX must be reserved for only a very select group of older patients.

### Neoadjuvant treatment

Currently, neoadjuvant treatment of patients with resectable tumours is not considered a standard treatment, so it must be carried out in the context of a clinical trial. With the development of more advanced imaging techniques, a growing number of patients are included in the subgroup of patients with borderline-resectable pancreatic cancer [32]. In this context, the indication for neoadjuvant treatment has gradually been incorporated into routine clinical practice, since it allows not only a reduction in tumour size and a greater likelihood of a complete resection [33], but also permits a better selection of candidates for surgery by identifying those who would progress on neoadjuvant treatment due to greater tumour aggressiveness and worse prognosis. This strategy is considered particularly attractive for older patients with borderline-resectable tumours, in many of whom the start of adjuvant chemotherapy can be delayed or even ruled out due to postsurgical complications or comorbidities.

Few studies have explored the role of neoadjuvant treatment in older patients. Miura et al. presented efficacy data in older patients (≥ 75 years old) with resectable and borderline-resectable tumours. Among the patients who completed treatment, there were no significant differences in complication rate or OS. The prognostic factors for treatment failure were borderline-resectable tumour (versus resectable tumour), elevated carbohydrate antigen 19.9 (CA 19.9) after neoadjuvant treatment, and high anaesthetic or surgical risk, but not age [34].

Regarding the treatment scheme of choice for neoadjuvant treatment, the greatest evidence in the general population comes from phase II trials and a meta-analysis with neoadjuvant FOLFIRINOX based mainly on retrospective studies [35–37]. In these studies, few patients are > 75 years old (in the meta-analysis, it is only mentioned that 50% are ≥ 60 years old), and in most, the percentage of resectability and survival data are similar to those obtained in younger patients. Therefore, age is not considered a poor prognostic factor, with surgical resection rates of approximately 60% (R0 83.9%), a PFS of 18 months, and an OS of 22.2 months [37]. With these data showing no differences in OS or resectability rates, it also seems appropriate to recommend neoadjuvant treatment in the older population.

The gemcitabine and nab-paclitaxel (gemcitabine-nab-paclitaxel, GNP) regimen is also effective and safe in older patients. Results of patients aged 70 years or older with resectable or locally advanced pancreatic cancer who have received GNP have been published. Neoadjuvant chemotherapy was well tolerated and resulted in high rates of R0 resection (58%) and OS (18 months). The authors conclude that neoadjuvant chemotherapy with GNP is a safe and effective treatment option for older patients with pancreatic cancer [38].

Regarding the choice of scheme, as in adjuvant therapy, the use of CGA is necessary to optimize the patient’s treatment plan. However, due to low representation of the older population in studies with FOLFIRINOX, the regimen of choice is GNP or gemcitabine monotherapy.

### Radiation therapy

The role of radiotherapy (RT) in borderline or resectable PDAC is controversial and its impact on survival has not yet been clarified. Local treatment with neoadjuvant radiotherapy can help achieve a higher rate of postsurgical free margins (R0) and therefore a lower local recurrence rate when combined with prior sequential chemotherapy treatment. However, two phase III trials have not obtained enough evidence to recommend it as a standard treatment in this context [39, 40].

In a recent retrospective study with over 12,000 PDAC patients aged ≥ 65 years performed between 2004 and 2018, 21% received RT [41]. Notably, the survival advantage of patients treated with RT was more significant in those aged between 65 to 80 years, with regional and distant stage cancer, with no previous surgery as well as those who receive chemotherapy (median OS: 14.0 vs. 11.0 months; HR 0.82, 95% CI: 0.77–0.87; *p* < 0.001). These results suggest that RT can improve the outcome of older patients with PDAC in certain subgroups of patients. However, further prospective studies are needed to confirm these findings.

Radiation therapy alone in older patients can only be considered a valid option if they have comorbidities that prevent the administration of chemotherapy or in resectable cases where the morbidities or frailty contraindicate surgery, but always after being discussed within the Tumour Multidisciplinary Committee.

## Locally advanced pancreatic adenocarcinoma

Locally advanced PDAC represents approximately 25–30% of cases at the time of diagnosis and is strictly considered an unresectable disease [42]. Standard treatment is based on systemic chemotherapy, which has shown better survival and tolerance than chemoradiotherapy [43]. Gemcitabine-based regimens obtain an OS of approximately 8 months in the older population [44]. Combined chemoradiotherapy treatment has not shown benefit in OS but a modest benefit in local control, so in many cases it is prescribed after systemic treatment, always after discussion by a multidisciplinary committee [45].

External chemoradiotherapy treatment is usually well tolerated, but its administration for 5–6 weeks is sometimes not possible in an older patient with a poor prognosis. In contrast, stereotactic body radiotherapy (SBRT) is sometimes more appealing and easier to sequence with systemic treatment, which is why it is considered an attractive therapy for the older population [46]. A recent study analysed the efficacy of SBRT (24 Gy/single fraction) alone or with chemotherapy in 26 patients ≥ 80 years old. The OS of the patients treated with SBRT was 7.6 months, without grade 3 toxicity. The treatment also achieved symptomatic improvement of abdominal or lumbar pain [47].

## Metastatic pancreatic adenocarcinoma

In recent decades, there have been significant advances in the treatment of PDAC. Given that the older population is underrepresented in clinical trials, treatment in this age group continues to pose a therapeutic challenge [48]. These uncertainties mean that patients ≥ 65 years old receive chemotherapy less frequently than those < 65 years old (52% vs. 74%) [49].

### First-line treatments

Currently, the standard first-line treatment for patients with metastatic PDAC is considered to be the combination of FOLFIRINOX or GNP (Table 3). In the phase III clinical trial PRODIGE 4/ACCORD 13, FOLFIRINOX was compared with gemcitabine monotherapy, and OS was longer in the FOLFIRINOX arm (11.1 vs. 6.8 months; HR 0.57; p < 0.001). There were many grade 3–4 toxicities, mainly neutropenia (51%), asthenia (23%), and diarrhoea (13%). Although patients up to 75 years of age were allowed, only 28% were between 65 and 75 years of age, and toxicity was not described in this age subgroup. Regarding efficacy, this subgroup benefited from treatment with FOLFIRINOX (HR 0.48), although age ≥ 65 years was identified as a poor prognostic factor (HR 1.47; 95% CI 1.07–2.02; p = 0.019) [50]. Some later, smaller retrospective studies have described administering FOLFIRINOX to patients ≥ 70 years old, confirming that both efficacy and toxicity do not change with age [51, 52]. The PAMELA70 clinical trial has recently been completed, the objective of which was to determine the efficacy and tolerability of FOLFIRINOX at an age-adjusted dose in older patients (NCT02143219). The results will help to position this scheme in the geriatric population. Finally, it should be noted that the PANOPTIMOX-PRODIGE35 trial showed that maintenance treatment with 5-FU and leucovorin after 4 months of FOLFIRINOX reduced toxicity without losing efficacy, so this strategy may be especially useful in the geriatric population [53].

**Table 3:** Selected studies for the chemotherapeutic treatment of metastatic pancreatic adenocarcinoma

Study N	Age	Treatment	OS	HR	P-value
First-line treatment					
Conroy et al*. *[50] Phase III, *N* = 342	< 65: 71% ≥ 65: 29%	A: FOLFIRINOX B: Gemcitabine	A versus B 11.1 versus 6.6 m	0.57	0.001
Von Hoff et al*. *[54] Phase III, *N* = 891	< 65: 58% ≥ 65: 42%	A: GNP B: Gemcitabine	A versus B 8.6 versus 6.7 m	< 65: 0.65 ≥ 65: 0.81	0.001
Hasegawa et al*. *[56] Phase II, *N* = 27	≥ 75	GNP	10.3 m	NR	NR
Feliu et al*. *[55] Phase II, *N* = 80	≥ 70	GNP	9.2 m	NR	NR
Second-line treatment					
Wang et al*.* [59] Phase III, *N* = 417	< 65: 54% > 65: 46%	A: NalIRI/FL B: FL	A versus B 6.1 versus 4.2 m	0.67	0.012

*FL* Fluorouracilo-Leucovorín, *FOLFIRINOX* leucovorin calcium (folinic acid), fluorouracil, irinotecan hydrochloride, and oxaliplatin, *GNP* gemcitabine-nab-paclitaxel, *HR* hazard ratio, *m* month, *NalIRI* Nal-irinotecán; *NR* not reported, *OS* overall survival

The MPACT trial compared the administration of GNP with gemcitabine monotherapy and showed a benefit in OS in favour of the GNP combination (8.5 vs. 6.7 months; HR 0.72; *p* < 0.001) [54]. The main grade 3–4 toxicities were neutropenia (38%), asthenia (17%), neuropathy (17%), and diarrhoea (6%). Age was not an inclusion criterion, so 42% were ≥ 65 years old and 10% were ≥ 75 years old. The group of patients ≥ 65 years old obtained the same benefit in terms of PFS as the younger group (HR 0.69), but their OS was lower (HR 0.81) [54]. Since then, several phase II trials have shown that this scheme is effective and well tolerated in older individuals [55, 56]. Modifications of the GNP scheme carried out in older individuals, such as omitting day 8 of the cycle, can decrease its effectiveness (OS 9.44 vs. 7.63 months; *p* = 0.003) [57]. On the other hand, maintenance treatment with gemcitabine after receiving three full cycles of GNP seems not to reduce the effectiveness of the treatment [58].

### Beyond first-line treatment

Second-line treatment for metastatic pancreatic cancer in the population ≥ 75 years of age is not well defined. Comorbidity, previous toxicity, and tumour progression usually cause most older patients with ECOG ≥ 2 to resort to second-line treatment, best supportive care being the recommended therapeutic alternative. In patients with good general and functional status (ECOG 0–1) who had progressed on a gemcitabine-based regimen, the phase III NAPOLI trial administered a second line of treatment with nanoliposomal irinotecan (nal-IRI), 5-FU, and leucovorin (NalIRI/5-FU/LV) [59].

The NAPOLI-1 study included patients with a Karnofsky performance status ≥ 70 who had previously received a gemcitabine-based regimen. The NalIRI/5-FU/LV scheme showed a benefit over 5-FU/LV in terms of OS (6.1 months vs. 4.2 months; HR 0.67; *p* = 0.012), PFS, and objective response rate. A later analysis of the older population of the NAPOLI-1 study (192 patients ≥ 65, 110 patients ≥ 70, and 43 patients ≥ 75) showed that this subgroup had risks of progression and death similar to the global population and similar toxicity (HR for OS/PFS was 0.88/0.95 in < 65 vs. ≥ 65 years old and 0.89/0.88 in < 70 vs. ≥ 70 years old).

Patients aged 65 to 75 years with good general and functional condition (ECOG 0–1) who have been treated with FOLFIRINOX as first-line treatment can receive the regimen of GNP or gemcitabine alone as second-line treatment. In that case, the NalIRI/FL/LV scheme would be indicated in patients with metastatic pancreatic cancer that has progressed after a previous line of treatment with a gemcitabine-based regimen.

**Fig. 2:**
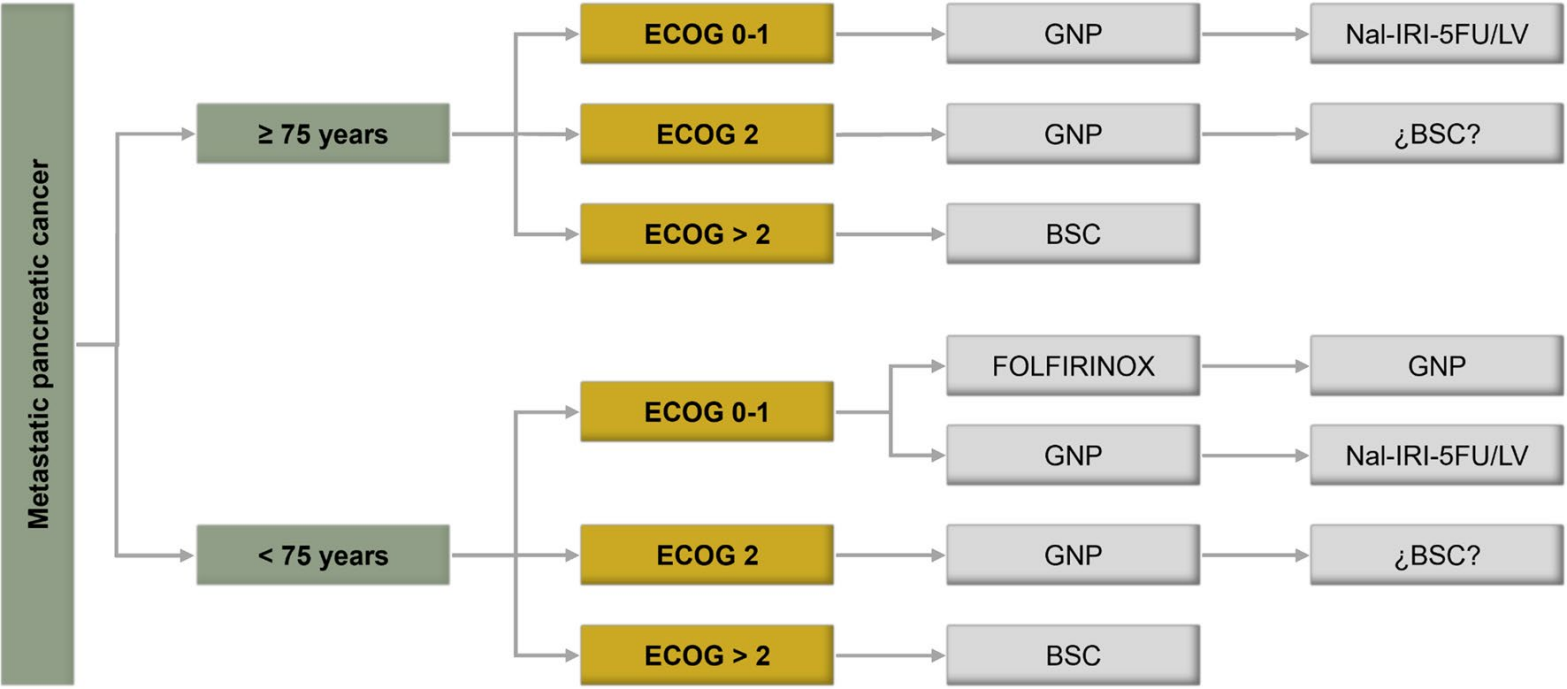
Therapeutic algorithm for older patients with metastatic pancreatic cancer. *BSC* best supportive care, *ECOG* Eastern Cooperative Oncology Group, *FOLFIRINOX* 5-fluorouracilo-leucovorín-Irinotecán-oxaliplatino, *GNP* gemcitabine-nab-paclitaxel, *Nal-Iri-5FU/LV* Nal-Irinotecán-5-fluorouracilo-leucovorín

In summary, the special characteristics of the older population and the fact that chemotherapy for metastatic pancreatic cancer is not curative make it necessary to carefully evaluate the potential benefits offered by the treatments in terms of quality of life, survival, symptomatic relief from any toxicity, and risk of aggravating a patient’s comorbidities. Taking these aspects into account, Fig. 2 proposes an algorithm for the management of these patients. Although no randomized clinical trials have evaluated the impact of CGA on the survival of older patients with pancreatic cancer, the information it provides is useful to identify patients who can tolerate antineoplastic treatment, so this should always be carried out before starting treatment.

## Targeted therapies and clinical research in older patients with pancreatic adenocarcinoma

Limited studies have focused on the treatment of older patients with PDAC. In 1999, an analysis of more than 16,390 patients included in clinical trials was published in the *New England Journal of Medicine*. The authors concluded that patients > 65 years old were underrepresented in clinical trials (25% vs. 65% younger patients). When pancreatic cancer patients were analysed, 73% of this population in the United States were > 65 years old, but only 38% of pancreatic patients in clinical trials were > 65. The authors commented that the low representation of older patients in clinical trials was due not only to patient and family factors but also to the oncologists themselves, who were especially reluctant to include them because of their comorbidities, which make them more complex patients who are less likely to meet the inclusion criteria of these studies, and because they expected these patients to have a higher incidence of toxicity associated with the new drugs, although there were no data to support such notions [60]. An added problem in this population is the greater number of drugs that they generally take. It is also harder to move older patients to the centres where these clinical studies are done. The authors found that in most of the studies analysed, there was actually no age limitation for inclusion.

Some years later, another analysis of 16,042 patients with pancreatic cancer included in 38 phase III clinical trials was published [61]. The median age in these studies was 62 years. Efficacy and age-related toxicity were only evaluated in two studies. In these, patients ≥ 75 years of age developed fatigue, thrombocytopenia, and infections more often than patients < 75 years of age, although the drug had the same efficacy in both groups. Since a significant proportion of patients with pancreatic cancer are older, efforts should be made so that the clinical studies that are carried out evaluate the efficacy and toxicity of treatments based on age. To do this, it would be of great help to use geriatric scales that evaluate the risk involved in the administration of certain treatments in older patients.

There is only one biomarker-supported treatment in pancreatic cancer: olaparib after induction therapy with platinum-based chemotherapy in patients carrying the germline mutation in the breast cancer gene 1 (*BRCA1*) or breast cancer gene 2 (*BRCA2*) [62]. The analysis of the POLO study according to the age of the patients showed that olaparib was effective at all ages, with a response rate of 27% (< 65 years old) compared to 15% (≥ 65 years old). Patients who maintained olaparib treatment for a period equal to or greater than 2 years had a similar response rate in both groups (11%), as did the toxicity profile. Nor were differences in quality of life detected according to the age of the patient [63].

Patients with pancreatic tumours with a nonmutated *KRAS* proto-oncogene have a higher incidence of potentially treatable alterations. This situation is more frequent in young patients (< 50 years old) and very rare in older patients, so in precision medicine, age is a disadvantageous factor [64]. In a study comparing a group of young patients (< 50 years old) with pancreatic cancer with a group with the usual age, the younger group had a higher proportion of the nonmutated *KRAS* gene than the older group (17% vs. 4%) [65]. In a study in which 2483 patients with pancreatic cancer were analysed, 11% of the tumours had nonmutated *KRAS*, and these tumours had a better prognosis and characteristics that favoured the administration of immunotherapy (higher percentage of microsatellite instability, greater tumour mutational burden, and greater infiltration of CD8^+^ inflammatory cells). Furthermore, these tumours had a greater number of potentially treatable therapeutic targets. The median age of this series was 66 years [66].

Data have been presented on zenocutuzumab, an inhibitor of human epidermal growth factor receptor 2 (HER2) and 3 (HER3), which has shown promising efficacy in patients with pancreatic cancer carrying the fusion in the neuregulin 1 (*NRG1*) gene. This alteration has been described almost only in patients with nonmutated *KRAS* who are under 50 years of age. The median age of the study patients was 49 years [67].

Therefore, for precision medicine, older age is a limitation because the proportion of tumours with nonmutated *KRAS* falls with age. The efficacy of the new drugs under investigation in patients with the *KRAS* mutation, in which age is not a limitation, remains to be seen [68].

## Supportive treatment

It is essential to improve the symptoms associated with PDAC in older patients and adjust the treatment to the expectations and quality of life of the patient. PDAC is sometimes characterized by intense symptoms, so their alleviation takes priority. Most of the complications are produced by the growth and infiltration of the tumour into neighbouring structures, which can cause biliary and gastrointestinal obstruction, pain, and, due to systemic symptoms, thromboembolic phenomena and anorexia-cachexia, among others.

### Management of biliary obstruction in older patients

Sometimes, the presenting symptom of PDAC is biliary obstruction, and in up to 70% of patients it appears at some point during the disease course [69]. Diagnosis by imaging tests shows a mass effect with bile flow obstruction, causing dilation of the intrahepatic bile duct [69].

**Fig. 3:**
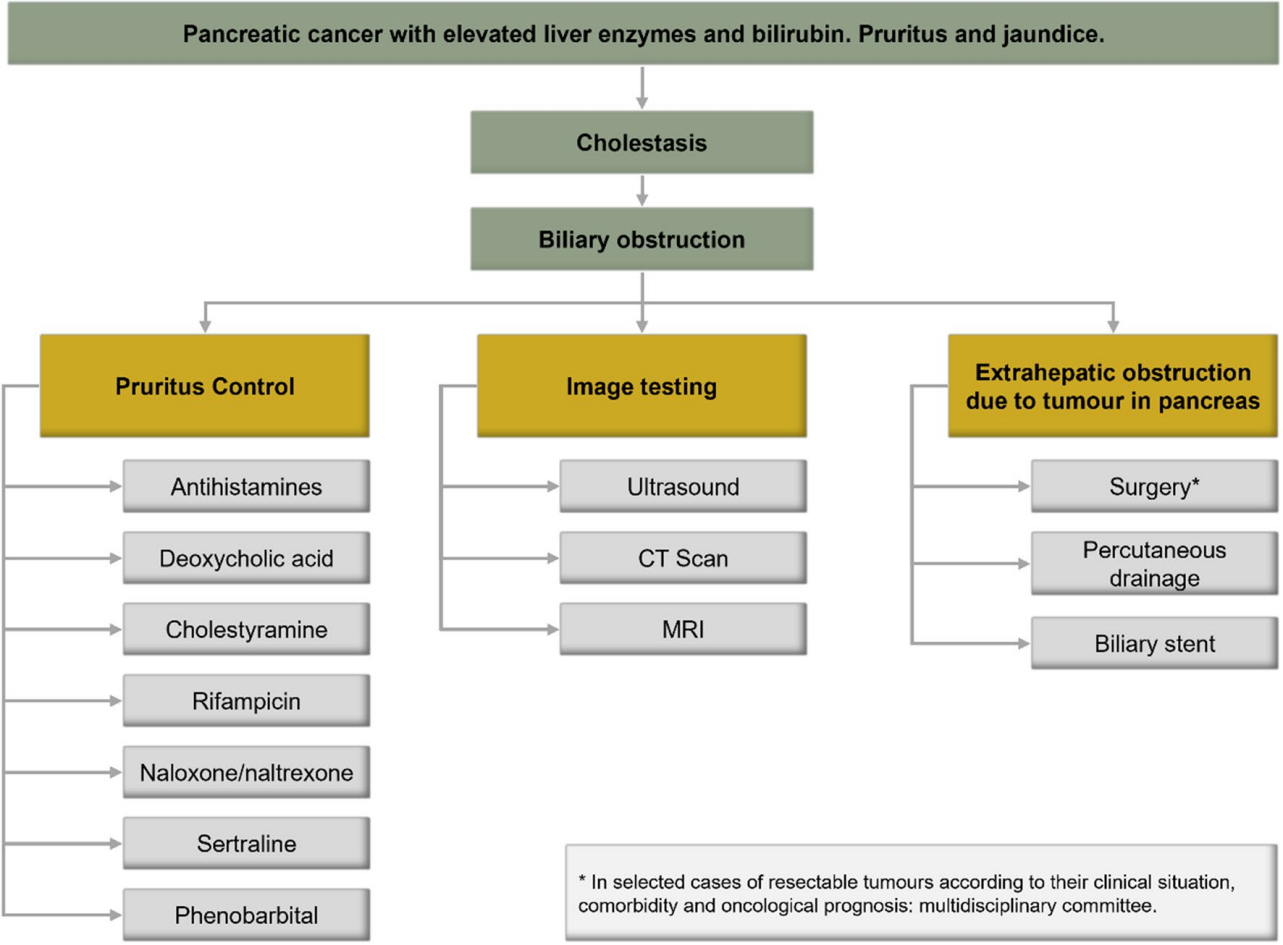
Biliary obstruction. *CT* computed tomography; *MRI* magnetic resonance imaging

Before treating obstructive jaundice due to pancreatic cancer, it is necessary to take into account previous comorbidities, the oncological prognosis, and the clinical situation of the patient. The main objective should always be the symptomatic relief of jaundice and the prevention of liver failure secondary to chronic obstruction [70]. Medical treatment aims at the control of itching, for which drugs such as antihistamines, ursodeoxycholic acid, cholestyramine, and other drugs less used such as rifampicin, naloxone/naltrexone, sertraline or phenobarbital [69]. Interventional treatment aims to unblock the bile by means of transhepatic or endoscopic biliary drainage [69]. Biliary stenting is the preferred interventional treatment. Endoscopic prostheses have the advantage of avoiding external drainage, which can be difficult to manage for an older patient. This technique is effective in more than 80% of cases and should be a first option for older patients with a reasonable life expectancy. Biliary bypass surgery may be considered when a stent cannot be placed. In certain patients with a resectable tumour and good functional status, absence of contraindications and after an assessment in the functional committee, surgery may be an option (Fig. 3).

Manipulation of the bile duct may be associated with an increased risk of cholangitis. Other complications can be bleeding, perforation, cholecystitis, and pancreatitis. In the case of the stent, occlusion or migration should be suspected if abdominal pain or elevated liver enzymes and hyperbilirubinemia are present [69].

In general, due to the prognosis of biliary obstruction, palliation using minimally invasive techniques is the most appropriate for most patients, especially older patients [70], to avoid invasive processes that bring more complications, longer periods of recovery, and more hospitalizations.

### Neoplastic pain

Neoplastic pain occurs due to involvement of the celiac plexus and is usually described as dull, insidious, or spasmodic. It is the most frequent presenting symptom in pancreatic cancer and arises in 70–100% of patients at some point during the disease [69]. For this reason, periodic evaluations and adequate pain management are crucial to improve the quality of life of these patients. In addition, poorly controlled pain leads to functional impairment, anxiety, depression, sleep disturbances, and anorexia [71].

In the case of older patients, special consideration must be given when assessing pain tolerance and the safety of opioids. Older patients are more sensitive to these drugs, so they must start with lower doses than usual and must have a longer interval between administrations. In addition, polypharmacy is common among older patients, and there may be interactions between drugs that exacerbate adverse effects or medical conditions such as kidney failure, which may cause a decrease in the clearance or metabolism of morphine, so that close monitoring is necessary [72]. Some opioids are not recommended in the older population, such as transdermal meperidine, methadone, and fentanyl, due to the increase in the half-life of their metabolism, which may lead to an increase in adverse events.

Invasive analgesic procedures, such as celiac trunk blockade, can be useful, achieving pain control in 70–90% of cases and allowing the reduction of opiates, but their effect does not last long, only about 2–3 months. Older patients can benefit from this technique, though they might present more episodes of hypotension as a side effect [73].

### Anorexia-cachexia syndrome

Malnutrition in cancer patients is related to cancer-related anorexia-cachexia syndrome (CACS), a complex metabolic syndrome characterized by a persistently increased basal metabolism that causes loss of weight and muscle mass, with or without loss of fat, and is commonly accompanied by anorexia, activation of the systemic inflammatory system, insulin resistance, and increased tissue protein turnover [74]. The proportion of patients who present weight loss at diagnosis is 15–40%, but it varies depending on the type and stage of cancer, the incidence being very high in advanced stages. In pancreatic cancer, CACS can be seen in 86% of patients [75], and more than 40% of patients with this neoplasm have severe weight loss during treatment [76].

Among 80 older patients (≥ 75 years) with metastatic PDAC treated with chemotherapy, 60% exhibited cachexia, and 61 (76%) showed signs of sarcopenia. Cachexia was linked to older age, poorer performance status, a higher neutrophil to lymphocyte ratio, inferior nutritional status, and shorter time to treatment failure (TTF) and PFS. Additionally, it was associated with an early treatment discontinuation, a reduced relative dose intensity of nab-paclitaxel, and a higher occurrence of grade 4 neutropenia in GNP-treated patients. In contrast, sarcopenia had a relatively limited impact on the clinical course of older patients with PDAC [77].

Nutritional assessment and support are important in the setting of localized, advanced, or metastatic pancreatic cancer. There is evidence that prehabilitation as part of multimodal management improves functional capacity and psychological well-being [78]. Its objective is to reduce complications and secondary events of the treatments, as well as to improve recovery and quality of life, with high-risk patients, such as older and frail patients, who benefit the most from this management. In a retrospective study of older patients (> 65 years old) with pancreatic cancer, loss of appetite and hypoalbuminaemia were correlated with increased mortality [79]. This could be explained by inflammatory mechanisms or hormonal imbalance [80].

Therefore, the interventions that should be carried out in this population are nutritional advice with adaptation of diet and physical exercise, assessing supplementation or artificial enteral or parenteral nutrition according to individual requirements, taking into account the oncological prognosis and the patient’s clinical situation [69]. The effect seems to be dose-dependent, although corticosteroids have shown an orexigenic effect similar in magnitude to that of megestrol acetate, but without a correlation with weight gain [81, 82]. Drug treatment for orexigenic purposes, such as megestrol acetate, is aimed at increasing appetite and weight, as well as improving quality of life [69]. In pancreatic cancer, the contribution of pancreatic enzymes should be considered in the case of exocrine pancreatic insufficiency secondary to surgical resection or pancreatic failure due to tumour infiltration, since these patients frequently suffer from malnutrition and sarcopenia secondary to malabsorption [69, 78].

### Prevention and management of thrombosis

Cancer patients have 6–7 times the risk of a venous thrombotic event than the general population. In pancreatic cancer and its treatments, incidence rates exceed 30% in certain studies. Thrombotic events are not exclusive to the onset of the disease but can occur throughout the entire process, and their appearance has been demonstrated as an independent negative prognostic factor.

Thromboprophylaxis should be evaluated in patients with intermediate-high risk (Khorana score ≥ 2) and who do not present a high risk of bleeding [83]. If performed, it would be especially indicated in the first 6 months after diagnosis when 66% of the events occur. Low-molecular-weight heparins, rivaroxaban, and apixaban are adequate and safe in the older population [84].

## Conclusions

PDAC is very common, especially in the older population. Compared to younger patients, the prognosis of older patients with pancreatic cancer is poorer, mostly because of a shorter life expectancy. Nevertheless, the effectiveness of treatments as well as the survival benefit obtained from chemotherapy might be potentially comparable [85]. The treatment of older patients is complex, as several factors, such as comorbidity, polypharmacy, high risk of frailty, and the physiological changes that occur during ageing, must be considered. Another key aspect to discuss with every patient and their families is the expected quality of life during the course of the disease.

In localized stages, both presurgical selection through CGA and the implementation of oncogeriatric interventions are necessary due to the severe comorbidity that surgery can cause. Surgery in specialized high-volume centres is not associated with increased mortality in this population, and adjuvant chemotherapy with gemcitabine-based regimens may be a good option in selected older patients. In metastatic disease, new chemotherapy regimens have shown greater benefit in the older population, although this population is underrepresented in published clinical trials, so the evidence is scarce. In patients < 75 years of age and in good general condition, FOLFIRINOX can be considered. In patients > 75 years of age, who tend to have greater frailty, less toxic regimens, such as GNP, are preferable. In all cases and due to the high symptomatic burden of this tumour, best supportive care through nutritional support and pain control is vital.

Future clinical trials need to include more older patients, so their participation should be encouraged. The development of prognostic molecular markers that demonstrate the benefit of treatment should also be an objective in the older population. There is only one approved biomarker-targeting treatment in pancreatic cancer, which is olaparib after platinum-based chemotherapy.

Finally, the incorporation of CGA tools in a multidisciplinary context is strongly recommended to individualize treatment, prevent toxicity, and improve the care of older patients with PDAC.

## Data Availability

Not applicable.

## References

[1] Sociedad Española de Oncología Médica S. Las cifras del cáncer en España 2023. 2023. p. 40.https://www.seom.org Accessed 15 Nov 2023

[2] Kim SY, Weinberg L, Christophi C, Nikfarjam M (2017). The outcomes of pancreaticoduodenectomy in patients aged 80 or older: a systematic review and meta-analysis. HPB (Oxford).

[3] Sukharamwala P, Thoens J, Szuchmacher M, Smith J, DeVito P (2012). Advanced age is a risk factor for post-operative complications and mortality after a pancreaticoduodenectomy: a meta-analysis and systematic review. HPB (Oxford).

[4] Barbas AS, Turley RS, Ceppa EP, Reddy SK, Blazer DG, Clary BM (2012). Comparison of outcomes and the use of multimodality therapy in young and elderly people undergoing surgical resection of pancreatic cancer. J Am Geriatr Soc.

[5] Gironés Sarrió R, Antonio Rebollo M, Molina Garrido MJ, Guillén-Ponce C, Blanco R, Gonzalez Flores E (2018). General recommendations paper on the management of older patients with cancer: the SEOM geriatric oncology task force’s position statement. Clin Transl Oncol.

[6] Rodriquenz MG, Negrete-Najar JP, Sam C, Sehovic M, Extermann M (2022). Assessment of the external validity of the National Comprehensive Cancer Network (NCCN) guidelines for pancreatic ductal adenocarcinoma in a population of older patients aged 70 years and older. J Geriatr Oncol.

[7] Castel-Kremer E, De Talhouet S, Charlois AL, Graillot E, Chopin-Laly X, Adham M (2018). An onco-geriatric approach to select older patients for optimal treatments of pancreatic adenocarcinoma. J Geriatr Oncol.

[8] Ngo-Huang A, Holmes HM, des Bordes JKA, Parker NH, Fogelman D, Petzel MQB, et al. Association between frailty syndrome and survival in patients with pancreatic adenocarcinoma. Cancer Med. 2019;8(6):2867–76. 10.1002/cam4.2157. 10.1002/cam4.2157 PMC6558581 31033241

[9] Dale W, Hemmerich J, Kamm A, Posner MC, Matthews JB, Rothman R (2014). Geriatric assessment improves prediction of surgical outcomes in older adults undergoing pancreaticoduodenectomy: a prospective cohort study. Ann Surg.

[10] Gebbia V, Mare M, Cordio S, Valerio MR, Piazza D, Bordonaro R (2021). Is G8 geriatric assessment tool useful in managing elderly patients with metastatic pancreatic carcinoma?. J Geriatr Oncol.

[11] Nishijima TF, Deal AM, Williams GR, Sanoff HK, Nyrop KA, Muss HB (2018). Chemotherapy toxicity risk score for treatment decisions in older adults with advanced solid tumors. Oncologist.

[12] Extermann M, Boler I, Reich RR, Lyman GH, Brown RH, DeFelice J (2012). Predicting the risk of chemotherapy toxicity in older patients: the Chemotherapy Risk Assessment Scale for High-Age Patients (CRASH) score. Cancer.

[13] Beinse G, Reitter D, Segaux L, Carvahlo-Verlinde M, Rousseau B, Tournigand C (2020). Potential drug-drug interactions and risk of unplanned hospitalization in older patients with cancer: a survey of the prospective ELCAPA (ELderly CAncer PAtients) cohort. J Geriatr Oncol.

[14] Feliu J, Pinto A, Basterretxea L, López-San Vicente B, Paredero I, Llabrés E (2021). Development and validation of an early mortality risk score for older patients treated with chemotherapy for cancer. J Clin Med.

[15] Gajra A, Klepin HD, Feng T, Tew WP, Mohile SG, Owusu C (2015). Predictors of chemotherapy dose reduction at first cycle in patients age 65 years and older with solid tumors. J Geriatr Oncol.

[16] Gooiker GA, Lemmens VE, Besselink MG, Busch OR, Bonsing BA, Molenaar IQ (2014). Impact of centralization of pancreatic cancer surgery on resection rates and survival. Br J Surg.

[17] Riall TS, Sheffield KM, Kuo YF, Townsend CM, Goodwin JS (2011). Resection benefits older adults with locoregional pancreatic cancer despite greater short-term morbidity and mortality. J Am Geriatr Soc.

[18] Bilimoria KY, Bentrem DJ, Ko CY, Stewart AK, Winchester DP, Talamonti MS (2007). National failure to operate on early stage pancreatic cancer. Ann Surg.

[19] Groen JV, Douwes TA, van Eycken E, van der Geest LGM, Johannesen TB, Besselink MG (2020). Treatment and survival of elderly patients with stage I-II pancreatic cancer: a report of the EURECCA Pancreas consortium. Ann Surg Oncol.

[20] Nassoiy S, Christopher W, Marcus R, Keller J, Weiss J, Chang SC (2023). Evolving management of early stage pancreatic adenocarcinoma in older patients. Am J Surg.

[21] Henry AC, Schouten TJ, Daamen LA, Walma MS, Noordzij P, Cirkel GA (2022). Short- and long-term outcomes of pancreatic cancer resection in elderly patients: a nationwide analysis. Ann Surg Oncol.

[22] Oettle H, Neuhaus P, Hochhaus A, Hartmann JT, Gellert K, Ridwelski K (2013). Adjuvant chemotherapy with gemcitabine and long-term outcomes among patients with resected pancreatic cancer: the CONKO-001 randomized trial. JAMA.

[23] Frakes JM, Strom T, Springett GM, Hoffe SE, Balducci L, Hodul P (2015). Resected pancreatic cancer outcomes in the elderly. J Geriatr Oncol.

[24] Nagrial AM, Chang DK, Nguyen NQ, Johns AL, Chantrill LA, Humphris JL (2014). Adjuvant chemotherapy in elderly patients with pancreatic cancer. Br J Cancer.

[25] Oettle H, Post S, Neuhaus P, Gellert K, Langrehr J, Ridwelski K (2007). Adjuvant chemotherapy with gemcitabine vs observation in patients undergoing curative-intent resection of pancreatic cancer: a randomized controlled trial. JAMA.

[26] Neoptolemos JP, Stocken DD, Bassi C, Ghaneh P, Cunningham D, Goldstein D (2010). Adjuvant chemotherapy with fluorouracil plus folinic acid vs gemcitabine following pancreatic cancer resection: a randomized controlled trial. JAMA.

[27] Neoptolemos JP, Palmer DH, Ghaneh P, Psarelli EE, Valle JW, Halloran CM (2017). Comparison of adjuvant gemcitabine and capecitabine with gemcitabine monotherapy in patients with resected pancreatic cancer (ESPAC-4): a multicentre, open-label, randomised, phase 3 trial. Lancet.

[28] Reni M, Riess H, O’Reilly EM, Park JO, Hatoum H, Saez BL, et al. Phase III APACT trial of adjuvant nab-paclitaxel plus gemcitabine (nab-P + Gem) versus gemcitabine (Gem) alone for patients with resected pancreatic cancer (PC): outcomes by geographic region. J Clin Oncol. 2020;38(15_Suppl):4515. 10.1200/JCO.2020.38.15_suppl.4515.

[29] Conroy T, Hammel P, Hebbar M, Ben Abdelghani M, Wei AC, Raoul JL (2018). FOLFIRINOX or gemcitabine as adjuvant therapy for pancreatic cancer. N Engl J Med.

[30] Conroy T, Castan F, Lopez A, Turpin A, Ben Abdelghani M, Wei AC (2022). Five-year outcomes of FOLFIRINOX vs gemcitabine as adjuvant therapy for pancreatic cancer: a randomized clinical trial. JAMA Oncol.

[31] Gómez-España MA, Montes AF, Garcia-Carbonero R, Mercadé TM, Maurel J, Martín AM, et al. SEOM clinical guidelines for pancreatic and biliary tract cancer. Clin Transl Oncol. 2021;23(5):988–1000. 10.1007/s12094-021-02573-1. 10.1007/s12094-021-02573-1 PMC8058005 33660222

[32] Varadhachary GR, Tamm EP, Abbruzzese JL, Xiong HQ, Crane CH, Wang H (2006). Borderline resectable pancreatic cancer: definitions, management, and role of preoperative therapy. Ann Surg Oncol.

[33] Lim KH, Chung E, Khan A, Cao D, Linehan D, Ben-Josef E (2012). Neoadjuvant therapy of pancreatic cancer: the emerging paradigm?. Oncologist.

[34] Miura JT, Krepline AN, George B, Ritch PS, Erickson BA, Johnston FM (2015). Use of neoadjuvant therapy in patients 75 years of age and older with pancreatic cancer. Surgery.

[35] Janssen QP, Buettner S, Suker M, Beumer BR, Addeo P, Bachellier P (2019). Neoadjuvant FOLFIRINOX in patients with borderline resectable pancreatic cancer: a systematic review and patient-level meta-analysis. J Natl Cancer Inst.

[36] Kondo N, Uemura K, Sudo T, Hashimoto Y, Sumiyoshi T, Okada K (2021). A phase II study of gemcitabine/nab-paclitaxel/S-1 combination neoadjuvant chemotherapy for patients with borderline resectable pancreatic cancer with arterial contact. Eur J Cancer.

[37] Yoo C, Lee SS, Song KB, Jeong JH, Hyung J, Park DH (2020). Neoadjuvant modified FOLFIRINOX followed by postoperative gemcitabine in borderline resectable pancreatic adenocarcinoma: a Phase 2 study for clinical and biomarker analysis. Br J Cancer.

[38] Oba A, Wu YHA, Lieu CH, Meguid C, Colborn KL, Beaty L (2021). Outcome of neoadjuvant treatment for pancreatic cancer in elderly patients: comparative, observational cohort study. Br J Surg.

[39] Katz MHG, Shi Q, Meyers JP, Herman JM, Choung M, Wolpin BM, et al. Alliance A021501: preoperative mFOLFIRINOX or mFOLFIRINOX plus hypofractionated radiation therapy (RT) for borderline resectable (BR) adenocarcinoma of the pancreas. J Clin Oncol. 2021;39(3_Suppl):377. 10.1200/JCO.2021.39.3_suppl.377.

[40] Versteijne E, van Dam JL, Suker M, Janssen QP, Groothuis K, Akkermans-Vogelaar JM (2022). Neoadjuvant chemoradiotherapy versus upfront surgery for resectable and borderline resectable pancreatic cancer: long-term results of the Dutch Randomized PREOPANC trial. J Clin Oncol.

[41] Cao BY, Wang QQ, Zhang LT, Wu CC, Tong F, Yang W (2023). Survival benefits and disparities in radiation therapy for elderly patients with pancreatic ductal adenocarcinoma. World J Gastrointest Oncol.

[42] Li D, Xie K, Wolff R, Abbruzzese JL (2004). Pancreatic cancer. Lancet.

[43] Chauffert B, Mornex F, Bonnetain F, Rougier P, Mariette C, Bouché O et al. Phase III trial comparing intensive induction chemoradiotherapy (60 Gy, infusional 5-FU and intermittent cisplatin) followed by maintenance gemcitabine with gemcitabine alone for locally advanced unresectable pancreatic cancer. Definitive results of the 2000–01 FFCD/SFRO study. Ann Oncol. 2008;19(9):1592–9. 10.1093/annonc/mdn281. 10.1093/annonc/mdn281 18467316

[44] Maréchal R, Demols A, Gay F, de Maertelaer V, Arvanitaki M, Hendlisz A (2008). Tolerance and efficacy of gemcitabine and gemcitabine-based regimens in elderly patients with advanced pancreatic cancer. Pancreas.

[45] Hammel P, Huguet F, van Laethem JL, Goldstein D, Glimelius B, Artru P (2016). Effect of chemoradiotherapy vs chemotherapy on survival in patients with locally advanced pancreatic cancer controlled after 4 months of gemcitabine with or without erlotinib: the LAP07 randomized clinical trial. JAMA.

[46] Herman JM, Koong AC (2014). Stereotactic body radiation therapy: a new standard option for pancreatic cancer?. J Natl Compr Canc Netw.

[47] Kim CH, Ling DC, Wegner RE, Flickinger JC, Heron DE, Zeh H (2013). Stereotactic body radiotherapy in the treatment of pancreatic adenocarcinoma in elderly patients. Radiat Oncol.

[48] Macchini M, Chiaravalli M, Zanon S, Peretti U, Mazza E, Gianni L (2019). Chemotherapy in elderly patients with pancreatic cancer: efficacy, feasibility and future perspectives. Cancer Treat Rev.

[49] Nipp R, Tramontano AC, Kong CY, Pandharipande P, Dowling EC, Schrag D (2018). Disparities in cancer outcomes across age, sex, and race/ethnicity among patients with pancreatic cancer. Cancer Med.

[50] Conroy T, Desseigne F, Ychou M, Bouché O, Guimbaud R, Bécouarn Y (2011). FOLFIRINOX versus gemcitabine for metastatic pancreatic cancer. N Engl J Med.

[51] Baldini C, Escande A, Bouché O, El Hajbi F, Volet J, Bourgeois V (2017). Safety and efficacy of FOLFIRINOX in elderly patients with metastatic or locally advanced pancreatic adenocarcinoma: a retrospective analysis. Pancreatology.

[52] Mizrahi JD, Rogers JE, Hess KR, Wolff RA, Varadhachary GR, Javle MM (2020). Modified FOLFIRINOX in pancreatic cancer patients age 75 or older. Pancreatology.

[53] Dahan L, Williet N, Le Malicot K, Phelip JM, Desrame J, Bouché O (2021). Randomized phase II trial evaluating two sequential treatments in first line of metastatic pancreatic cancer: results of the PANOPTIMOX-PRODIGE 35 trial. J Clin Oncol.

[54] Von Hoff DD, Ervin T, Arena FP, Chiorean EG, Infante J, Moore M (2013). Increased survival in pancreatic cancer with nab-paclitaxel plus gemcitabine. N Engl J Med.

[55] Feliu J, Jorge Fernández M, Macarulla T, Massuti B, Albero A, González González JF (2021). Phase II clinical trial of nab-paclitaxel plus gemcitabine in elderly patients with previously untreated locally advanced or metastatic pancreatic adenocarcinoma: the BIBABRAX study. Cancer Chemother Pharmacol.

[56] Hasegawa R, Okuwaki K, Kida M, Yamauchi H, Kawaguchi Y, Matsumoto T (2019). A clinical trial to assess the feasibility and efficacy of nab-paclitaxel plus gemcitabine for elderly patients with unresectable advanced pancreatic cancer. Int J Clin Oncol.

[57] Winer A, Handorf E, Dotan E. Dosing schedules of gemcitabine and nab-paclitaxel for older adults with metastatic pancreatic cancer. JNCI Cancer Spectr. 2021;5(5). 10.1093/jncics/pkab074. 10.1093/jncics/pkab074 PMC8438244 34532641

[58] Petrioli R, Torre P, Pesola G, Paganini G, Paolelli L, Miano ST (2020). Gemcitabine plus nab-paclitaxel followed by maintenance treatment with gemcitabine alone as first-line treatment for older adults with locally advanced or metastatic pancreatic cancer. J Geriatr Oncol.

[59] Wang-Gillam A, Li CP, Bodoky G, Dean A, Shan YS, Jameson G (2016). Nanoliposomal irinotecan with fluorouracil and folinic acid in metastatic pancreatic cancer after previous gemcitabine-based therapy (NAPOLI-1): a global, randomised, open-label, phase 3 trial. Lancet.

[60] Sehgal R, Alsharedi M, Larck C, Edwards P, Gress T (2014). Pancreatic cancer survival in elderly patients treated with chemotherapy. Pancreas.

[61] White MN, Dotan E, Catalano PJ, Cardin DB, Berlin JD (2019). Advanced pancreatic cancer clinical trials: the continued underrepresentation of older patients. J Geriatr Oncol.

[62] Golan T, Hammel P, Reni M, Van Cutsem E, Macarulla T, Hall MJ (2019). Maintenance olaparib for germline BRCA-mutated metastatic pancreatic cancer. N Engl J Med.

[63] Kindler H, Hammel P, Reni M, Cutsem EV, Macarulla T, Hall M (2020). SO-3 Maintenance olaparib in patients aged ≥65 years with a germline BRCA mutation and metastatic pancreatic cancer: phase III POLO trial. Ann Oncol.

[64] Aguirre AJ, Nowak JA, Camarda ND, Moffitt RA, Ghazani AA, Hazar-Rethinam M (2018). Real-time genomic characterization of advanced pancreatic cancer to enable precision medicine. Cancer Discov.

[65] Ben-Aharon I, van Laarhoven HWM, Fontana E, Obermannova R, Nilsson M, Lordick F (2023). Early-onset cancer in the gastrointestinal tract is on the rise-evidence and implications. Cancer Discov.

[66] Philip PA, Azar I, Xiu J, Hall MJ, Hendifar AE, Lou E (2022). Molecular characterization of KRAS wild-type tumors in patients with pancreatic adenocarcinoma. Clin Cancer Res.

[67] Schram AM, O’Reilly EM, O’Kane GM, Goto K, Kim D-W, Neuzillet C, et al. Efficacy and safety of zenocutuzumab in advanced pancreas cancer and other solid tumors harboring NRG1 fusions. J Clin Oncol. 2021;39(15_Suppl):3003. 10.1200/JCO.2021.39.15_suppl.3003.

[68] Bekaii-Saab TS, Spira AI, Yaeger R, Buchschacher GL, McRee AJ, Sabari JK, et al. KRYSTAL-1: updated activity and safety of adagrasib (MRTX849) in patients (Pts) with unresectable or metastatic pancreatic cancer (PDAC) and other gastrointestinal (GI) tumors harboring a KRASG12C mutation. J Clin Oncol. 2022;40(4_Suppl):519. 10.1200/JCO.2022.40.4_suppl.519.

[69] De las Peñas... et al. 3ª edición Manual SEOM de Cuidados continuos. Madrid: Gonext Producciones SL; 2019.

[70] Siegel JH, Kasmin FE (1997). Biliary tract diseases in the elderly: management and outcomes. Gut.

[71] Ferrell BR, Wisdom C, Wenzl C (1989). Quality of life as an outcome variable in the management of cancer pain. Cancer.

[72] Sheehan DC, Forman WB (1997). Symptomatic management of the older person with cancer. Clin Geriatr Med.

[73] Russell RC (1999). Palliation of pain and jaundice: an overview. Ann Oncol.

[74] Tuca A, Jimenez-Fonseca P, Gascón P (2013). Clinical evaluation and optimal management of cancer cachexia. Crit Rev Oncol Hematol.

[75] Chow R, Bruera E, Chiu L, Chow S, Chiu N, Lam H (2016). Enteral and parenteral nutrition in cancer patients: a systematic review and meta-analysis. Ann Palliat Med.

[76] Del Olmo García MD, Ocón Bretón J, Álvarez Hernández J, Ballesteros Pomar MD, Botella Romero F, Bretón Lesmes I (2018). Terms, concepts and definitions in clinical artificial nutrition. ConT-SEEN Project Endocrinol Diabetes Nutr (Engl Ed).

[77] Takeda T, Sasaki T, Suzumori C, Mie T, Furukawa T, Yamada Y (2021). The impact of cachexia and sarcopenia in elderly pancreatic cancer patients receiving palliative chemotherapy. Int J Clin Oncol.

[78] Bibby N, Griffin O. Nutritional considerations for the management of the older person with hepato-pancreatico-biliary malignancy. Eur J Surg Oncol. 2021;47(3 Pt A):533–8. 10.1016/j.ejso.2020.04.024. 10.1016/j.ejso.2020.04.024 32362465

[79] Ohta R, Moriwaki Y, Sano C (2022). Association between survival duration of older patients with advanced unresectable pancreatic cancer and appetite loss: a retrospective cohort study. Healthcare (Basel).

[80] Shadhu K, Xi C (2019). Inflammation and pancreatic cancer: an updated review. Saudi J Gastroenterol.

[81] Loprinzi CL (1995). Management of cancer anorexia/cachexia. Support Care Cancer.

[82] Loprinzi CL, Kugler JW, Sloan JA, Mailliard JA, Krook JE, Wilwerding MB (1999). Randomized comparison of megestrol acetate versus dexamethasone versus fluoxymesterone for the treatment of cancer anorexia/cachexia. J Clin Oncol.

[83] Khorana AA, Rao MV (2007). Approaches to risk-stratifying cancer patients for venous thromboembolism. Thromb Res.

[84] García Adrián S, González AR, de Castro EM, Olmos VP, Morán LO, Del Prado PM (2022). Incidence, risk factors, and evolution of venous thromboembolic events in patients diagnosed with pancreatic carcinoma and treated with chemotherapy on an outpatient basis. Eur J Intern Med.

[85] Jung HA, Han BR, Kim HY, Kim HJ, Zang DY, Jung JY (2021). Treatment and outcomes of metastatic pancreatic cancer in elderly patients. Chemotherapy.

[86] Fried LP, Tangen CM, Walston J, Newman AB, Hirsch C, Gottdiener J (2001). Frailty in older adults: evidence for a phenotype. J Gerontol A Biol Sci Med Sci.

